# Editorial: Insights Into Brown Adipose Tissue Functions and Browning Phenomenon

**DOI:** 10.3389/fphys.2020.00219

**Published:** 2020-03-06

**Authors:** Paula Oliver, Assunta Lombardi, Rita De Matteis

**Affiliations:** ^1^Nutrigenomics and Obesity Group, University of the Balearic Islands, Palma, Spain; ^2^Health Research Institute of the Balearic Islands (IdISBa), Palma, Spain; ^3^CIBER de Fisiopatología de la Obesidad y Nutrición, Madrid, Spain; ^4^Department of Biology, University of Naples Federico II, Naples, Italy; ^5^Department of Biomolecular Sciences, University of Urbino Carlo Bo, Urbino, Italy

**Keywords:** thermogenesis, mitochondria, uncoupling proteins (UCPs), obesity, metabolic disease, adipocytes, dietary components

Originally identified as part of the thermogenic system in mammals, researchers were quick to perceive the potential of brown adipose tissue (BAT) to increase energy expenditure and to control energy homeostasis. Experimental evidence regarding the thermogenic capacity of BAT made it a plausible therapeutic target for inducing weight loss, increasing energy expenditure, and contributing to whole-body energy balance.

The recent discovery of functional BAT in humans has generated enormous interest, based on the already clear acceptance that the tissue is present, thermogenically active, and exhibits plasticity. The ability to recruit so-called brite/beige adipocytes within white adipose tissue (WAT) has further added to the focus on BAT. WAT browning represents a particularly intriguing concept in humans given the extreme amount of excess WAT in obese individuals. Moreover, the obesity pandemic and its metabolic complications have focused attention on adipose tissue and the molecular mechanisms underlying energy homeostasis, raising important questions concerning the metabolic proprieties of thermogenic fat.

This Research Topic aims to provide a thorough overview of research progress on BAT biology, exploring unexpected putative roles for brown/brite cells, and presenting new activators and physiological conditions promoting the browning process.

The Opinion article of Trayhurn provides a comprehensive historical overview of the key role of BAT thermogenesis in the control of energy homeostasis and identifies the viability of therapeutic applications for obesity treatment, in the light of human metabolism. Although substantive evidence points to BAT activation and browning as interesting obesity therapeutic strategies, there are also substantive arguments against the realistic usefulness of this therapeutic approach in humans. Thus, in spite of the fact that active BAT contributes to glucose uptake and triglyceride clearance, its contribution to total energy expenditure is as yet not clear.

In this line, conflicting data about the contribution of BAT to cold-induced thermogenesis (CIT) in humans raise the question of the importance of BAT as a realistic therapeutic target to induce weight loss. The provocative original paper of Sanchez-Delgado et al. describes the effect of a personalized cold protocol on young adults. Contrary to what could be expected, their data show no contribution of either BAT or muscle activity to CIT or to cold-induced nutrient oxidation. Lack of a physiological plausible association could be due to experimental variability/technological limitations for human metabolism quantification, which is an important limitation in thermogenesis research. However, these results could also point to a marginal role of BAT in human CIT and, therefore, the energy metabolism.

Meanwhile, beyond their controversial role in energy waste and body weight control, it is now evident that brown/brite cells are involved in new functions that could transform the perspective of the tissue and its potential therapeutic application. Some contributions to this knowledge are published in this Research Topic. In recent years, in research mainly performed in rodents, browning of periaortic adipose tissue has been linked to cardiovascular protection. Reynés, van Schothorst et al. analyse cold response in adipose tissue of ferrets, a model closer to humans than rodents, and demonstrate clear adipose remodeling specifically in perivascular in comparison to subcutaneous fat. This browning induction along with the decreased expression of immune-related genes could be relevant for cardiovascular protection.

Moreover, the new concept of “metabolically active” BAT suggests that the beneficial effects of brown/brite activation could be due to UCP1 independent mechanisms, in both physiological and pathological conditions, associated with enhanced mitochondrial oxidative machinery. In the review of Pohl et al. the spotlight is focused on the emerging yet still enigmatic role played by UCP3 in BAT, the only tissue that simultaneously expresses two homologous proteins, with a similar proton transport rate but with UCP3 expression being 400-fold less than UCP1. The review also focuses on the molecular mechanism by which purine nucleotides differently inhibit UCP1 and UCP3, and on the direct correlation between UCP3 abundance and degree of cell fatty acid oxidation.

In addition, the emerging concept of a specific brown fat secretoma provides a physiologically significant link between adipose tissue, systemic metabolism, and health status. In this Research Topic, Villarroya et al. describe the brown adipocyte secretome in a cell culture medium in response to cAMP to mimic thermogenic activation. Surprisingly, most of the up-regulated secreted proteins correspond to extracellular matrix components, which could therefore play a key role in BAT remodeling. Unexpectedly, complement-related proteins are also secreted, probably contributing to signaling properties mediated by brown adipocytes. All the functions carried out by these secreted proteins are worth exploring in order to understand the metabolic roles of BAT. In this regard, Neuregulin 4 is a batokine that has become clinically relevant as a marker of metabolic health in murine models. Comas et al. show that, in humans, Neuregulin 4 may be considered a novel browning marker as its expression in adipose tissue correlates with that of brown/brite adipocyte markers and insulin action.

All in all, recent discoveries highlight the relevance of research concerning the physiological and pharmacological tools promoting brown/brite appearance of fat to thwart both excessive fat accumulation and consequent metabolic dysregulation.

The chronic effects of high-fat eating are more difficult to ignore, as an interesting article of Kuipers et al. describes in this Research Topic. In both white and brown fat of mice, the intake of a high-fat diet rapidly increases the synthesis pathways and circulating levels of endocannabinoids, which can have autocrine and even paracrine effects on adipose tissue by inhibiting noradrenergic signaling through activation of the G-protein-coupled receptor CB1R. Activation of CB1R decreases lipolysis in WAT and thermogenesis in BAT, leading to a greater positive energy balance and contributing to the development of obesity. Therefore, selective inhibition of CB1R on brown adipocytes or inhibition of endocannabinoid synthesis appears as a potential anti-obesity strategy.

Another important topic is nutritional programming during pregnancy. The article of Albustanji et al. mainly focuses on the impact of housing temperature on fat mass gain during pregnancy, and confirms the confounding effect of temperature on the outcome of metabolic studies. The obesogenic capacity of diets at different housing temperatures may contribute to an understanding of the mechanisms by which diet influences obesity development during pregnancy, which is reflected in adipose tissue features (adipogenesis) and metabolic alterations in the pups.

An obesogenic environment contributes to the whitening of brown fat and to the pathological processes associated with obesity. This may influence the correct differentiation/recruitment of BAT through different processes, such as catecholamine resistance, inflammation, oxidative stress, and endoplasmic reticulum stress, as described in the review article by Alcalá et al..

On the other hand, the innovative article of Asnani-Kishnani et al. demonstrates that intake of specific food bioactives in early postnatal life can influence the fate of preadipocytes in WAT toward a brown-like adipogenesis transcriptional program. Particularly, the authors analyse the role of resveratrol and of the vitamin B3 form nicotinamide riboside orally administered to post-natal mice. These results point toward metabolic programming which could program a protection to developmental obesity, with sexual dimorphism.

Meanwhile, there is a case for emerging obesogenic stimuli coming from the environment. In this context, Di Gregorio et al. point attention to the opposite effects elicited by different environmental pollutants on body energy homeostasis and BAT thermogenic activity.

Unfortunately, the modern lifestyle puts pressure on the homeostatic system responsible for the regulation of body weight and metabolism. Redirecting nutritional, environmental, and behavioral (daily) habits could help us discover new physiological stimuli to unlock the therapeutic potential of brown/brite fat. For this purpose, three intriguing reviews are presented in this Research Topic: Srivastava and Veech present evidence from recent literature concerning the regulation of BAT activity and browning by endocrine factors, dietary- and phyto-derived components. The effect of physical exercise on BAT activity and browning is also analyzed, pointing toward the divergent response elicited by rodents and humans. Furthermore, Madsen et al. suggest that high protein diets may modulate the energy metabolism, including the possible role of activating brown and brite adipocytes, futile cycles, and UCP1-independent mechanisms. Intriguing data from rodent and human trials are discussed, pointing attention to the lack of consistence when comparing both species. Last but not least, Reynés, Palou et al. suggest a key link between prebiotics and the regulation of adaptive thermogenesis and lipid metabolism (in both BAT and WAT). Thus, a connection is established between prebiotic consumption, microbiota selection (especially gut microbiota), production of microbiota metabolites, and regulation of the energy metabolism in adipose tissue, particularly regarding the effects on browning promotion, or on BAT recruitment.

This emerging dietary- and metabolite-mediated control of BAT activity and WAT browning could occur either directly or indirectly through specific modulation of traditionally noradrenergic regulation via β-adrenergic signaling. Adrenergic agonists are commonly considered to be inductors of WAT and BAT thermogenesis. In their original article, Clookey et al., by using a rodent model of menopause associated metabolic dysfunction [estrogen receptor alpha (ERα) null mice], show that adrenergic activation mitigates the metabolic dysfunction associated to the loss of ERα signaling through WAT browning and UCP1 induction.

On the other hand, Schnabl et al. reveal novel non-adrenergic activators of brown adipocytes. They identify six non-adrenergic targets to activate and recruit UCP1 in BAT. Among these ligands, the adrenocorticotropic hormone is described as one of the most potent activators of respiration and UCP-1 dependent thermogenesis in adipocytes, suggesting that, in physiological situations, it could mediate an acute effect of stress on BAT.

An important player in WAT browning induction is FGF21. The review of Cuevas-Ramos et al. describes the molecular pathways of browning induced by FGF21 and the UCP1-dependent and independent mechanisms by which FGF21 integrates metabolic pathways to regulate human energy balance and metabolism.

Finally, the review of Ro et al. focuses on the relevance of a proper regulation of autophagy in obesity prevention. In fact, obesity has been associated to autophagic dysregulation in adipose tissues. Therefore, as reviewed by the authors, an unexplored manipulation of the autophagic pathways could be considered in order to regulate WAT browning and adipogenesis reprogramming.

## Conclusions

Beyond the controversial contribution of BAT/browning to human whole-body energy expenditure, the new face/aspects presented in this Research Topic confirm the unequivocal evidence of the benefits conferred by the promotion of brown/brite phenotype of adipose tissue and give further evidence for its therapeutic role. Taking into consideration the available information, brown/brite adipocytes emerge as key players of metabolic homeostasis; therefore, new therapeutic perspectives, based on drugs, diet compounds/dietary and behavioral habits, and not necessarily focused on UCP1 activation, should be considered. Finally, a more profound understanding of the underlying mechanisms involved in browning promotion of energy homeostasis, which remains poorly understood, would help design health-promoting therapies.

## Author Contributions

All authors have made a substantial, direct and intellectual contribution to the work, and approved it for publication.

### Conflict of Interest

The authors declare that the research was conducted in the absence of any commercial or financial relationships that could be construed as a potential conflict of interest.

